# The Role of Sperm Morphology Standards in the Laboratory Assessment of Bull Fertility in Australia

**DOI:** 10.3389/fvets.2021.672058

**Published:** 2021-05-26

**Authors:** V.E.A. Perry

**Affiliations:** ^1^Queensland Sperm Morphology Laboratory, Ruminant Reproduction Research Centre, Goondiwindi, QLD, Australia; ^2^School of Veterinary Science, University of Queensland, Brisbane QLD, Australia; ^3^Robinson Research Institute, University of Adelaide, Adelaide, SA, Australia

**Keywords:** sperm morphology laboratory assessment standardization, bull fertility correlation, sperm laboratory standardization, correlation of fertility with sperm abnormalities, bull

## Abstract

The lack of standardization in the laboratory assessment of semen questions the reliability of semen analysis, and makes meaningful interpretation of these evaluations impossible. We herein describe a standardization program for morphology assessment currently in place in Australia and outline the methods used, both for the categorization of the abnormalities, including newly described abnormalities, and those that permit standardized microscopic assessment between laboratories.

## Introduction

The determination of the percentage morphologically normal bull sperm is highly repeatable and is strongly correlated with days to conception and calf output in both dairy herds (1–3) and beef herds (4). Increasingly, however, bull semen, in collection centers, is often evaluated only for total sperm numbers (concentration) and sperm motility (3). Furthermore, flow cytometry and measures of membrane intact sperm are replacing microscopy due to the faster turn over of these techniques (5).

The lack of standardization of laboratory semen assessment, including morphology, is highlighted by Brito (6). He observed significant differences in classifications and results in studies conducted between eight semen processing centers, laboratory and processing centers and five veterinary University laboratories. He concluded that: These observations question the reliability of semen analysis and make it impossible to meaningfully interpret evaluation.

The term “morphology assessed” may also be abused as it can be performed using improper protocols. For example, examination of sperm under low power microscopy with the inability to detect important abnormalities The ASMA (automated computer assisted sperm morphology analysis) tool for example can increase standardization but its inability to report all but basic head measurement and surface features means that many abnormalities are undetected (7) with little relationship with fertility reported (8).

The importance of a standardized approach led to the Australian standardization UQSMSP (University of Queensland Sperm Morphology Standardization Program) developed in 2018. It furthered the standardized morphology assessment reported in 2006 (9), as part of the standardized BBSE to be used by the Australian Cattle Veterinarian (ACV). This scheme uses central laboratories to provide unbiased expert analysis of sperm morphology (10–13). The progress of this standardization scheme along with advances in our knowledge of bull sperm abnormalities is reported here.

## Discussion

In the US, Canada and the UK morphology examination is usually completed crush side using vital stains such as nigrosin eosin which enable assessment of morphology under bright field microscopy. This method has been shown to be less accurate in its assessment of morphological abnormalities, particularly head abnormalities, in many studies (1, 14–17) when compared to the assessment of wet mounts under DIC (Differential Interference Contrast) or phase contrast microscopy usually completed in a specialized laboratory.

The considered professional gold standard for both the assessment of bovine and equine sperm morphology is DIC microscopy at x1,000 magnification, the recommended standard for Australian laboratories. Samples are sent to the central laboratories in buffered formal saline, which enables high quality wet preparations to be examined by the morphologist. Even with this level of microscope it is still necessary to focus up and down on each sperm to accurately assess abnormalities at the limit of resolution. This, however, is less the case than is necessary with Phase contrast microscopy (13). In recent years the advance of the DIC microscope has enabled even difficult to detect abnormalities of the DNA e.g., pale centers, to be viewed without the aid of Feulgen staining, although the latter is instigated as a base check.

**Figure 1:**
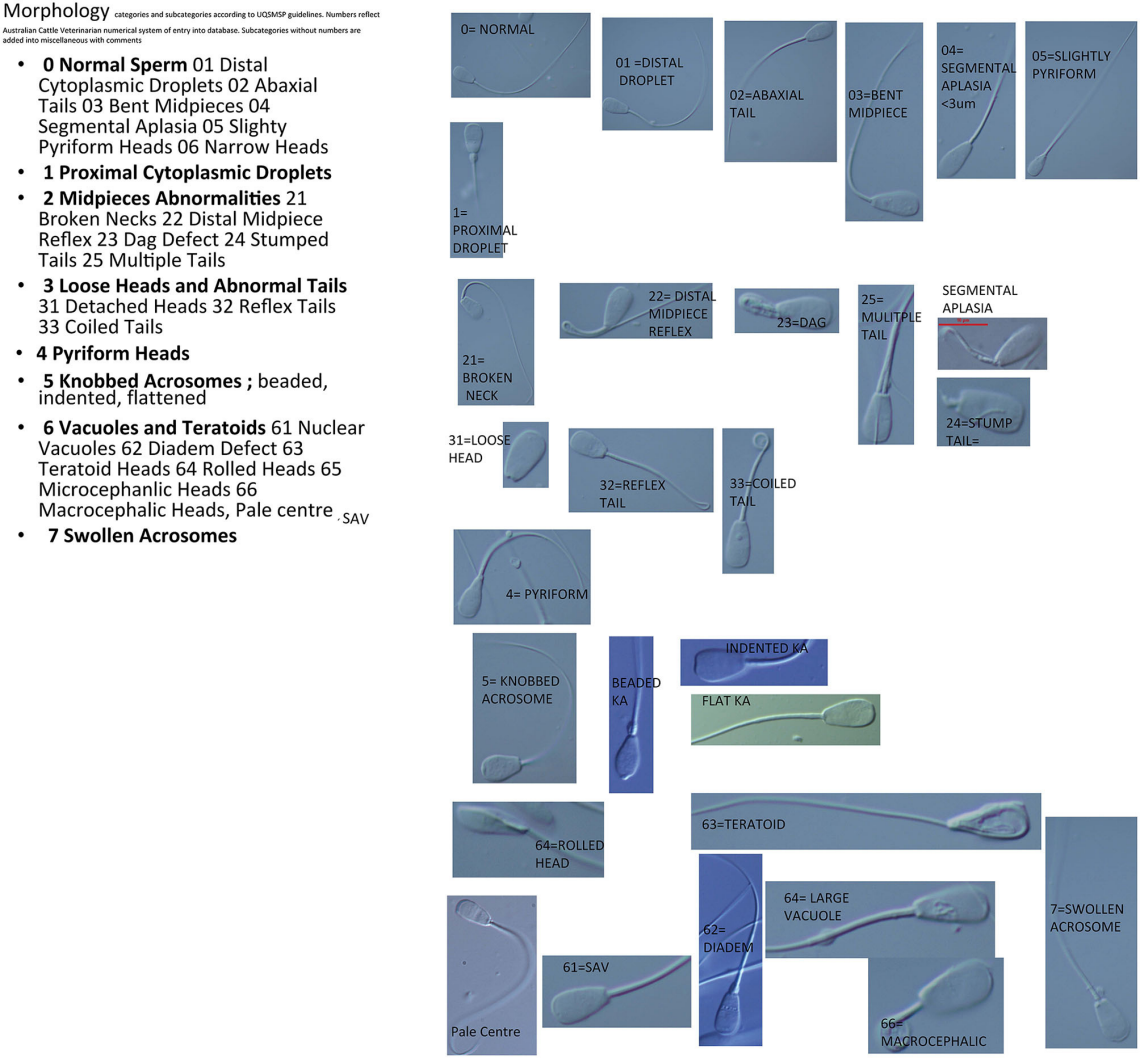
Images of sperm defects as categorized for the UQSMSP.

Fertile bulls have a spermiogram, which contains <30% abnormal sperm (4, 18, 19). This threshold level is accepted as standard in Australia as in many other countries, importantly, however, individual thresholds for each abnormality (10) vary and are based upon the currently known effect upon fertility. Each defect on each abnormal sperm is recorded; that is, more than one defect may be recorded per sperm. This is important in a standardized program as one morphologist may count a different abnormality to another. All counts are completed using the online morphology counting system developed by the ACV. This system allocates numbers to each trait (Figure 1). These numbers are entered into the keyboard and the software collates the number of abnormalities which creates the morphology report.

Morphologists are required to update their skills at an annual workshop and implement skills which maximize both the number and randomization of fields of view, e.g., only sperm in the middle of the field of view are counted. A minimum of 100 sperm are counted per spermiogram (12). This is however, increased to 200 in the case of borderline (62–77) counts (20).

Evenson (21) divided sperm into those with compensable or uncompensable traits. This concept is simplified if we consider that the female tract (22) and finally the vestments of the ovum act as a filtration system for the sperm population. They act as barriers to the progress of sperm such that only the fittest arrive at the ovum (23). Compensable traits preclude affected sperm from fertilizing the ovum, i.e., the abnormality does not allow them either to reach the ova or attach to the ova. A compensable abnormality, therefore, is one that can be compensated for by increasing the number of spermatozoa in the ejaculate; that is the fertility of the bull will increase with increasing numbers of spermatozoa. These include traits, which cause, for example, abnormal or nil motility (these are filtered out in the female tract), and abnormal head shape (filtered out crossing the zona as these interfere with hyperactive motility required at this juncture). The threshold for such abnormalities is set at 30% (12, 24, 25). Increasing numbers of spermatozoa, however, cannot compensate for uncompensable traits. Sperm with these traits are able to reach the ovum and initiate fertilization (thereby blocking polyspermy) and/or embryo development but that development is unsustainable. The cow therefore returns to oestrus. Such traits include, nuclear vacuoles and pyriform heads. They tend to be the subtler more difficult to detect abnormalities yet cause the biggest decrease in conception rates. The suggested threshold of such abnormalities is therefore 20% (12, 25). In general it could be said that sperm with abnormalities that do not allow them to reach the ova or attach are considered compensable traits. Those abnormalities, which allow the sperm to fertilize the ova but result in early embryonic death or abnormal development, are considered uncompensable.

UQSMSP issues guidelines on the equipment and protocols required to be upheld by members. The eight main sperm categories with tolerance levels are, in order; normal sperm-(which includes abnormalities observed but that are considered not to effect conception rates), proximal droplets (PD) (20%), midpiece abnormalities (MP) (30%), loose heads and principle piece (tail) abnormalities (HT) (30%), pyriform heads (PY) (20%), knobbed acrosomes (KA) (30%), vacuoles and teratoids (VT) (including abnormalities of DNA condensation) (30%), swollen acrosomes (SA) (including those sperm with lost acrosomes) (30%) (9). These main categories are further divided into sub categories in the advanced sheet view, for example; differentiation between flat acrosomes, beaded acrosomes and indented knobbed acrosomes, and the various categories of vacuolation (13). All morphologists must complete their counts using this advanced sheet with all subcategories to enable comparisons between morphologists if any queries arise on the counts completed.

The maintenance of standardization is emphasized by the requirement that samples are kept by morphology laboratories for 3 years and that these samples were made available to UQSMSP examiners where a disagreement arises. Each morphologist is required to perform competency checks on five samples per annum which are sent out from UQSMSP and results submitted back for analysis within 2 weeks. Unacceptable variation from the median results in second round of test samples being completed with the option of additional tuition supplied by UQSMSP.

When conducting the assessment of the spermiogram the morphologists must be cognisant of both the stages of spermatogenesis as well as the environmental and developmental effects upon sperm morphology. It is established that any environmental stress sufficient to cause elevation in circulating cortisol is sufficient to affect sperm morphology (4, 26). A cascade of linked developmental processes occur during spermiogenesis that are testosterone dependent, such as;formation of the acrosome from the golgi apparatus, compaction of sperm chromatin, mitochondrial and centriole organization to form the flagellum and initiation of cytoplasm resorption. This is neatly shown in the publication by Callaghan et al. (27) where a single acidotic event was followed sequentially by elevated cortisol, reduced FSH and testosterone consequent with increased sperm abnormalities in the subsequent weeks. Similar sequential appearance of spermatozoal abnormalities were observed following transport and relocation (13) or dexamethasone (28).

Analogous abnormalities have been observed following exposure to heat whether due to obesity, scrotal abnormality, climate or fever (29). Mechanisms that maintain testis homeothermy include the cremaster and dartos muscles and the testicular vascular cone (30). This latter consists of the coiled veins of the pampiniform plexus and the incoming testicular artery. A distinct scrotal neck is necessary for the adequate functioning of the testicular vascular cone, where heat exchange occurs between the venous and arterial blood. This may be absent in the obese animal. The testis are particularly susceptible to heat as testicular function occurs in a marginally hypoxic environment where an increase in temperature may increase metabolic rate, but there is no corresponding increase in blood flow. Tissues are therefore susceptible to hypoxia (28). Alternately, a detailed study (31) of testicular blood flow in sheep and mice recently suggests that it is heat itself rather than hypoxia that affects testicular function. A recent study, (32), found the number of bulls passing the sperm morphology test at 70% were reduced in Far Northern Australia, although, climatic region had less effect than breed. Equally season may affect morphology either via temperature or nutritional intake (11) with one study showing an elevation in bulls failing the knobbed acrosome and vacuole thresholds in the summer months (32).

Nutritional deficiencies during development, whether prenatal (33), pre weaning (34–36) or pre sale (27, 37, 38) have been shown to affect maturation of the spermiogram. During adulthood nutritional restriction and/or dietary change may have deleterious effects particularly in bulls predisposed to developing certain sperm abnormalities such as nuclear vacuoles (27). Immature spermiograms in pubertal and peri pubertal bulls display particularly high levels of proximal droplets which may vary between ejaculates collected on the same day (33, 38).

Dietary intake of toxic substances such as gossypol in cotton seed has been shown to affect morphology in some studies (39) but not in others (40). The dietary supply of metallic cations (e.g., calcium, iron) is thought to cause this differential effect as these bind gossypol in the rumen (40) and may be present in, for example, the mineral content of bore water or when lime is added to the diet.

These environmental effects overlay inherited conditions such as the knobbed acrosome (41–43), and the Dag defect (44). Relatedly breed has a significant effect upon morphology: For example the Belgian blue compared to the Friesian (45) and Bos indicus breed bulls compared to Bos taurus breeds (32). A Canadian study (46), however, reported no effect of breed on the spermiogram between the Bos taurus breeds used.

## Standardization Under UQSMSP

Images of each category are given in Figure 1.

### Normal Sperm

This category includes normal variations of form and those sperm with abnormal forms that are recognized as having no effect upon fertility in the bull. This includes: abaxial tail, minor segmental aplasia, distal droplets, slightly bent midpiece, slightly pyriform. The reasoning behind their inclusion as normal is listed below.

### Proximal Droplets

These are normally observed in the pubertal bull with incidence decreasing with age (33, 47). In the mature bull they indicate abnormal spermiogenesis (and/or epididymal function). They were observed 7–10 days following a temperature or stress event (28) and 15 days following ruminal acidosis (27).

The prognosis depends upon the type of abnormalities associated with the proximal droplets. Counts of 10–15% proximal droplets (35) have been associated with decreased fertility. This trait is considered uncompensable as the sperm fail to bind to the ova and furthermore that sperm associated with high numbers of proximal droplet sperm have impaired ability to bind with the ova (48). Amann et al. (47) also reported that in bulls with >30% proximal droplets that the associated apparently normal spermatozoa displayed immaturity and reduced ability to fertilize ova. PDs are also associated with decreased membrane integrity and increased chromatin damage post-thaw (49). This defect has a threshold of 20% as studies show proximal droplets are associated with poor pregnancy rates (50).

### Distal Droplets

Unlike the boar there are no reports of distal droplets being associated with infertility in the bull. Sperm with distal droplets will lose the droplet if left in a water bath for 15–30 min or if gently agitated. The number of sperm within the ejaculate with distal droplets, also vary widely between sequential ejaculates (12). Case studies using bulls with high numbers of distal droplets in natural service achieve normal pregnancy rates (51). For this reason distal droplets in isolation are not generally considered to be a defect by the author or by other researchers (24, 51) and are placed in the normal category.

### Cause

The Sertoli cell effects elongation of the spherical spermatid from stage 8–12 along with the exertions of the manchette (52). The surplus cytoplasm and organelles from this process remain attached to the spermatid as a residual body attached at the sperm neck. All sperm entering the caput epididymis therefore, have this droplet, however, only 10% remain by the time sperm leave the cauda epididymis The presence of a cytoplasmic droplet whether in the proximal or distal position may be an indication that the sperm has not acquired essential binding proteins from the seminal vesicle fluid (53). These binding proteins are essential for the sperm to bind to the zona pellucida. For this reason it is important that massage of the ampullae and seminal vesicles is sufficient to illicit a quantity of seminal fluid during the collection process (12).

### Pyriform Heads

Narrow in the postacrosomal region. Young bulls up to 2 years old and in good condition display a greater likelihood of recovery from this condition than do older bulls. This condition is particularly seen in young over fat bulls (51). It is very important to note, that there is variation in the degree of this abnormality: In a series of experiments Barth et al. (54) reported that fertility was related to the severity of pyriformity of the head. A moderate degree of pyriformity, in the absence of other signs of disturbed spermatogenesis, is not detrimental to fertility. However, extreme tapering in the postacrosomal region results in significant reductions in fertility. Pyriformity is considered only partially compensable (55). As in this study some pyriform sperm were able to fertilize oocytes but these had a reduced ability to cleave. The threshold of not more than 20% is therefore applied to this abnormality.

### Cause

Pyriform heads are induced in bulls following stress such as dexamethasone treatment and scrotal insulation (28) 20d post insult with some bulls showing predisposition to this abnormality following a stress event (51). Pyriform heads are differentially excluded from advancement in the female reproductive tract at the specifically precluded from the cervix, uterus, utero-tubule junction (22).

### Knobbed Acrosomes

This abnormality may be heritable or arise following a stress event (27) and is often observed in the peripubertal bull (33, 38) prior to the adult spermiogram. It was observed to rise 30 d after a single acidotic event (27).

Two forms are regularly observed; beaded and indented, however a third form; flattened, a subcategory of the indented form (56, 57) is essential to differentiate due to the fertility prognosis of each.

The beaded form is considered inherited by an autosomal recessive gene (41, 42). The beaded form is often associated with sterility and usually occurs as a high percentage of the ejaculate.

The indented form is described as an enlargement of the apical ridge that then folds back on the apex of the sperm head and is much more common than the beaded form. In the pig this form is also associated with gene aberrations on chromosome 15 (43).

As stated, indented or flattened acrosomes vary in their effect upon fertility. In non-competitive matings such bulls may achieve near normal fertility however this may reflect that normal sperm coexisting with these sperm are in sufficient numbers to achieve conception as sperm with the flattened or indented form were unable to penetrate the zona pellucida (56). This abnormality is therefore considered a compensable defect and is given a 30% threshold.

This is supported by work by Andersson et al. (58) who reported that when present in <25% of sperm there was no decrease in fertility. In bulls with a high percentage of this abnormality (>80%) the indented acrosome defect may not be compensable as in such sperm did not bind to the zona pellucida and other sperm present in the ejaculate that appeared normal could bind to the zona the resulting zygotes had a reduced ability to cleave (59).

### Cause

**Figure 2:**
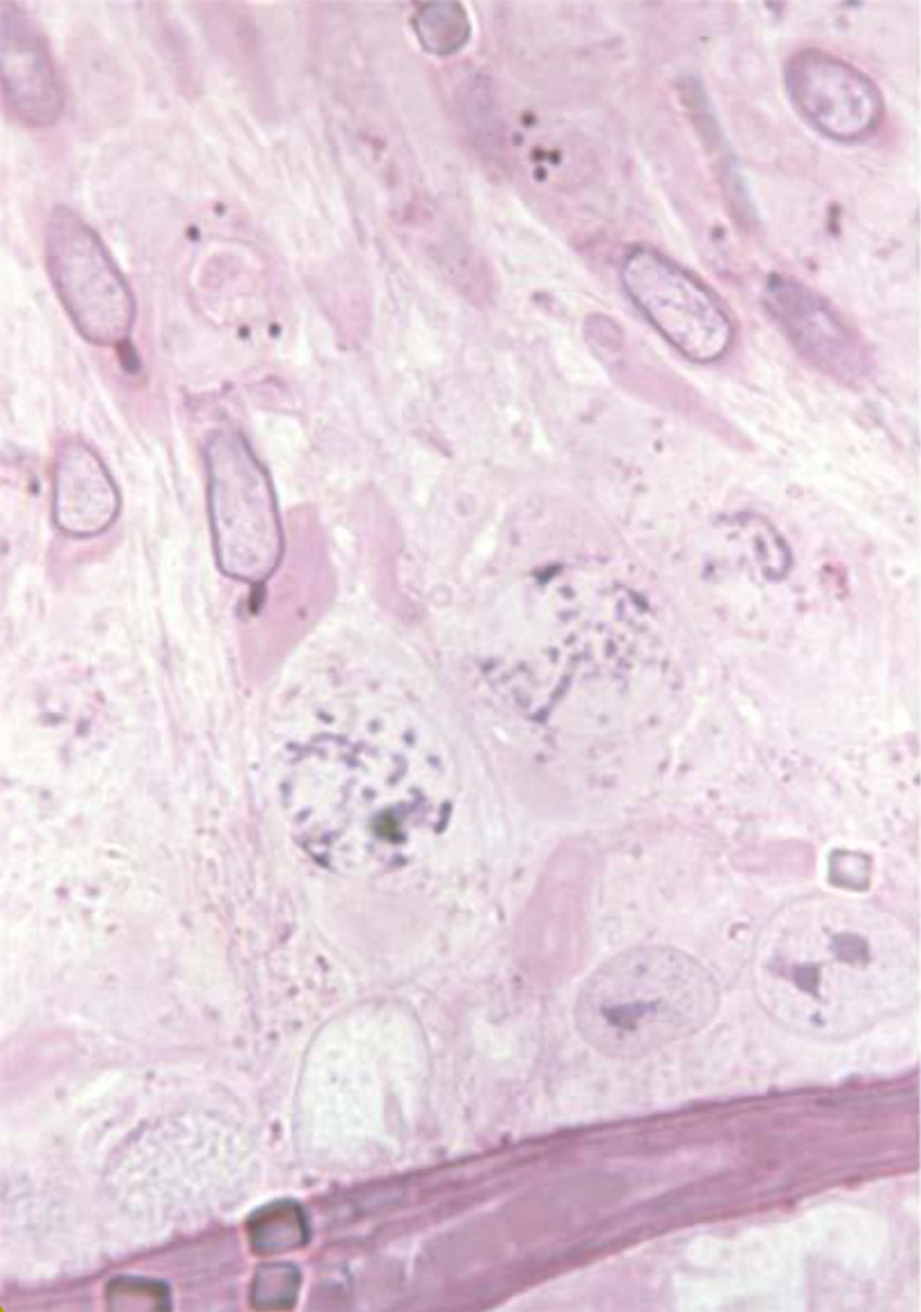
Images of the developing knobbed acrosome defect. Courtesy of Professor Albert Barth.

The acrosome develops from the Golgi-Endoplasmic reticulum system in the very early spermatid with the knobbed acrosome defect observed from the stage 7 spermatid (Figure 2). In the pig the KA defect has been linked to genes associated with ubiquitination; a prerequisite for both chromatin remodeling and acrosome formation (43). The KA defect is also actively selected against in the female reproductive tract such that normal sperm within the ejaculate are more likely to reach the ova (22).

### Swollen Acrosomes

These are given a separate category to knobbed as swelling and sloughing of the acrosome is a normal progression during sperm aging. The problem can be associated with “rusty load/ accumulated sperm” (12). Aging of the sperm causes the acrosome to undergo a similar reaction to capacitation resulting in the lifting of the acrosome, such sperm will be unable to attach to the oocyte. Swollen acrosomes are often seen in conjunction with other head abnormalities such as knobbed acrosomes. This is because the KA abnormality causes premature initiation of the acrosome reaction (59). The swollen acrosome in these cases may hide the knobbed defect in initial observations. In these cases spermiogenesis has obviously been disrupted. This abnormality is compensable and seldom occurs in very high numbers except in accumulators or when examining frozen thawed sperm.

### Vacuoles and Teratoids

Nuclear vacuolation occurs during spermiogenesis and may be caused be caused by an environmental stress although some there is some evidence for the heritability of this trait in some bulls (27, 32). The abnormality is more commonly observed in Bos indicus cross bulls than in Bos taurus breeds (32). Some bulls are predisposed to this condition (perhaps due to a hormone imbalance in the testis) following a stress event (27, 51). Vacuoles appeared 30 d after acidotic (27) event or 20 d after dexamethasone treatment (28). Three forms of vacuolation occur; large confluent vacuoles, diadem defect, small apical vacuoles.

Large confluent vacuoles (LCV) or craters can be so large as to be a “bite” size piece missing from the side of the head. This abnormality may occur after disruption to spermiogenesis, for example following a ruminal acidosis (27). It has also been reported as an inherited abnormality in a Santa Gertrudis herd (60). Smaller craters were also seen on other sperm in the ejaculate. Bulls with a high percentage of this abnormality were infertile. Canadian studies concur with this effect finding levels of >20% reduce pregnancy rates (51).

Apical vacuoles are commonly associated with the diadem defect or with multiple small vacuoles scattered throughout the nucleus. Unlike LCV or diadem however, they appear to be more transient than the other forms. Ejaculates with high numbers of apical vacuoles (80%) have reduced conception rates and in an IVF study no sperm with these vacuoles were observed inside the zona pellucida (51).

The diadem defect, an arrangement of vacuoles along the equatorial region of the sperm appears a serious cause of infertility in the bull. Fluctuations in the prevalence of this defect occurs between ejaculates (61) with stress being a predisposing factor.

A high incidence of the vacuole defect, >60% (62, 63) is known to cause severe reduction in fertility. There is debate as to whether this abnormality is compensable as some sperm with this defect could bind to the zona and did initiate fertilization (but it could not be determined if this fertilization produced viable zygotes) (63). Further, most of the sperm with this abnormality did not reach the zona. However, Pilip et al. (62) reported that sperm with multiple nuclear vacuoles had a reduced ability to fertilize ova.

In view of this uncertainty nuclear vacuoles are given a threshold level of not more than 20% (59).

### Abnormal DNA Condensation

This abnormality can be detected by SCSA- a flow cytometric assay that uses the metachromatic properties of acridine orange to measure the susceptibility of chromatin to denaturisation (29) or by Feulgen staining under x1,000 phase contrast or DIC microscopy. Feulgen and SCSA methods correlate and both enable assessment of affected sperm (64). Advances in DIC microscopy also now permit the examination of abnormal DNA condensation to a limited extent. When this or pale centers are considered the major reason for bull sperm falling below threshold Feulgen staining is often completed as a check.

### Pale Centers

Analogous to the DNA condensation this abnormality may be observed under Feulgen staining at x1,000 phase (or DIC) as the gold standard. However, it may also be observed under good DIC at x1,000. The abnormality is displayed as a narrowing of the sperm head in the PAS region. This abnormality has been reported to cause decreased fertility in a case study in Canada (Barth pers com) and in Australia by the author. This condition is currently under study by the author and colleagues. The abnormality may appear in conjunction with vacuole abnormalities or as the only abnormality present.

### Rolled Head Nuclear Crest-Giant Head Syndrome

This abnormality is also included under the vacuole/teratoid category as it is uncommon. It is thought to be an inherited condition. The prognosis for recovery is very poor (65). The number tolerated in the ejaculate is at 20% because of the ability to penetrate the zona pellucida but the inability to produce a viable embryo. Reports upon its effect, when present at 20–30% of the ejaculate, on conception rates vary between 27 and 74% (51).

### Teratoid Sperm

These are sperm that are so grossly abnormal in structure as to be barely recognizable as a sperm cell. The sperm nucleus varies from normal to grossly misshapen, may be vacuolated and the tail is often coiled up completely and lies superimposed on the head. These cells are indicative of severe disturbance to spermatogenesis and spermiogenesis. They often occur at very low levels in the spermiogram (1%) but when seen at higher levels the prognosis is poor. There should be no more than 15% of this type of sperm in an ejaculate and they should be associated with at least 70% normal sperm.

### Multinuclear - Multiflagellar Sperm Defect

Multiflagellar sperm are sometimes observed but this abnormality where the sperm have multiple nuclei, no acrosome and multiple tails has not been reported again in bulls to the authors knowledge (66).

### Midpiece Defects

#### Distal Reflex Midpieces

This is the most common defect seen in bull ejaculates (10, 46) not to be confused with a simple bent tail as the midpiece is also involved in the bend. This defect can occasionally arise as an artifact due to prolonged contact with a hypotonic solution (e.g., Nigrosin-Eosin stain), cold-shock, or solutions >pH 7. It is one of the first abnormalities to appear after a stress event as it occurs in the cauda epididymis [16 days after an acidotic event (27) or only 4–11 days after dexamethasone treatment (28)].

It is usually of a transient nature with recovery likely within 16 days. The presence of a cytoplasmic droplet at the tail bend identifies the problem as one occurring mainly in the distal half of the cauda epididymis. The prognosis varies with circumstance and the types of other abnormalities present. Where it occurs with abnormalities such as a fracture at the tail bend, aplasia of the midpiece or Dag-like defects there may be an underlying cause such as disturbed spermiogenesis. Some bulls have a predisposition for this defect with fluctuations in the percentage of affected sperm throughout the year. Up to 30% of this abnormality is tolerated in the ejaculate as these cells display reverse motility and would therefore be unable to penetrate the zona pellucida so other normal cells would be able to participate in ovum fertilization (65).

#### Dag-Like Defect

This can be an inherited defect with a serious effect upon fertility when present in large numbers (>50%) (65, 67). It can reflect disturbance in the testis or epididymis and is not normally present at >4%. It is a compensable trait as the sperm are not forwardly motile (25). Fertility is therefore only impaired once >30% of this defect is identified in the ejaculate with <70% normal sperm. Presences of fractured axonemal elements, with filaments protruding from the sheath are observed.

#### Segmental Aplasia of the Mitochondrial Sheath and the Pseudodroplet

In a case study bull with 90% segmental aplasia was reported to have normal fertility over 3 breeding seasons (29). This would indicate that the condition has little effect on fertility. This condition can be permanent or transient; if the defect is seen to occur in two tests done 10 weeks apart it suggests a permanent condition. Gossypol in the diet (39) and a viral disease (Bovine Ephemeral fever) (68), have both been shown to have an affect on the mitochondrial sheath. If gaps in the midpiece are larger than 3 microns these may result in fractures of the midpiece and sperm showing such severe segmental aplasia are considered under midpiece abnormalities, however, sperm observed with slight gaps are considered under the normal category (12).

A report of a new abnormality of the mitochondrial sheath which did not affect bull fertility (69) is similar to the previously described pseudodroplet both under light microscope and TEM images (51, 65, 70). The difference between the observations is that the effect upon the mitochondrial sheath is apical (69) compared to points along the midpiece (51) and that, at least in the Blom study this defect did affect fertility via an effect on motility of the sperm. The defect is observed as a thickening of the midpiece often associated with a bend or fracture. TEM reveals an accumulation of dense granules within these thickened areas. The light microscope images of the apical defect are similar to a broken neck appearance.

#### Abaxial Tails

The prognosis for this abnormality is determined by the presence or absence of an accessory tail. Ejaculates containing 60–100% spermatozoa with abaxial tails alone (71) cause no decrease in fertility. However, abaxial tails seen in an ejaculate with other spermatozoa with accessory tails (72) can cause a significant drop in fertility. The cause of this difference lies in the formation of the tail within the spermatid. Tail formation begins with the migration of the proximal and distal centriole to the base of the nucleus. The distal centriole gives rise to the tail with the proximal centriole forming the neck of the midpiece. Normally in spermatids replication of the centrioles is suppressed so that one flagella is formed. Lack of this suppression may allow the formation of additional tails. The presence of additional fossa and/or tails therefore may indicate the presence of additional centrioles. These structures are critical to the separation of chromosomes during the first cleavage of the ovum. This being the case, abaxial tails should not be considered a defect if present on their own. However, if abaxial tails are present at relatively low numbers (12–20%) with >17% accessory tails the bull would be considered of questionable fertility (65). Abaxial tails with accessory tails are considered within midpiece defect category, however, within the normal category if present on their own.

#### Tail Stump Defect

This condition is hereditary inherited via a recessive gene and has a poor prognosis. It is a compensable defect, as the sperm cannot journey to the fertilization site, bulls with 30–40% of this defect have been found to be fertile. It should be noted that care should be taken to differentiate this from detached heads as a cytoplasmic droplet often covers the vestigial midpiece portion.

### Loose Heads/Tail Abnormalities

#### Loose/Detached Heads

This is a problem that can arise with testicular degeneration or hypoplasia, inflamed ampullae or epididymis, heat stress and more usually, as a sign of a “rusty load.” If the motility is low in the initial crush side motility assessment of the semen then further ejaculates (up to 3) should be taken so that sperm that may have “accumulated” in storage can be eliminated and a representative sample collected. In the representative sample, fertility can be related to the percentage of detached heads found: the bull can still be considered “fertile” with 30–40% of this defect, but if the ejaculate contains 70% of this abnormality the bull would have severely decreased fertility. This is considered to be a minor abnormality and some latitude is allowed as it is considered to be a compensable effect; these sperm cannot participate in fertilization, as they cannot swim up the female tract.

#### Decapitated Head

The decapitated head defect has been reported in Guernsey and Hereford bulls. This may be an inherited problem. It can be differentiated from detached loose head by the large number of vigorously moving tails in the fresh specimen and the presence of the proximal droplet still attached to the tail. This trait when it occurs affects 80–100% of sperm in the ejaculate.

#### Principal Piece/Tail Defects

These are seldom seen in high numbers and may be caused by temperature shock or stress event during passage through the epididymis (28), therefore levels of this defect may decrease after 8–11 days. Levels of 30% are acceptable with 70% normal sperm as this is a compensable abnormality.

## Conclusion

It is important to firstly establish what we consider to be normal when we examine the ejaculate. We accept that a fertile bull should be >70% normal (4, 18, 19), however, this figure should be interpreted according to the type of abnormalities contained within the sample (9, 12, 29). Simply listing all of the abnormalities present is not helpful in forming a prognosis. Analagous to this, the laboratory should have the ability to give a prognosis based upon their knowledge of spermatogenesis together with information such as that provided by a full bull breeding soundness examination where environmental stressors are recorded (e.g., vaccination history, puberty, age, body condition etc.).

This standardized Australian model (9) has enabled increased accuracy of prognosis for practitioners and is well-regarded both in Australia and overseas (73, 74). The updated 2018 UQSMSP standardization scheme involving skill updates and training of morphology laboratories along with annual examination of work should result in reliable analysis which is easily interpreted.

## Author Contributions

The author confirms being the sole contributor of this work and has approved it for publication.

## Conflict of Interest

The author declares that the research was conducted in the absence of any commercial or financial relationships that could be construed as a potential conflict of interest.
